# Lung transplant after long-term veno-venous extracorporeal membrane oxygenation: a case report

**DOI:** 10.1186/s13019-021-01614-8

**Published:** 2021-08-30

**Authors:** Nobuyuki Yoshiyasu, Masaaki Sato, Masaki Anraku, Shingo Ichiba, Jun Nakajima

**Affiliations:** 1 Department of Thoracic Surgery, The University of Tokyo Hospital, 7-3-1 Hongo, Bunkyo-ku, Tokyo 113-8655 Japan; 2 Department of Surgical Intensive Care Medicine, Nippon Medical School Hospital, 1-1-5 Sendagi, Bunkyo-ku, Tokyo 113-8603 Japan

**Keywords:** Extracorporeal membrane oxygenation, Long-term bridging, Bone marrow transplantation, Chronic graft-versus-host disease, Lung transplantation

## Abstract

**Background:**

Although the number of patients who undergo extracorporeal membrane oxygenation (ECMO) as a bridge to lung transplantation is increasing worldwide, there are few reports on lung transplantation after long-term ECMO (more than 1 month). We report a rare case of successful bilateral lung transplantation in a Japanese patient after 5 months of veno-venous (VV)-ECMO support.

**Case presentation:**

A 27-year-old man who underwent bone marrow transplantation (BMTx) with fully matched human leukocyte antigen typing was diagnosed with bronchiolitis obliterans caused by chronic graft-versus-host disease 3 years after the BMTx. One year later, his respiratory condition had exacerbated, with carbon dioxide retention that required conventional mechanical ventilation. He was then deemed a suitable candidate for lung transplantation by a multidisciplinary transplantation selection committee. While waiting for donor lungs, his hypercapnia and acidosis became barely manageable under care with mechanical ventilation and ultimately he was switched to VV-ECMO. He remained on VV-ECMO for the next 5 months, during which period the circuit was switched nine times. In addition, sophisticated intensive care was required to manage multiple episodes of sepsis and coagulopathy. A suitable donor was identified 5 months later, and bilateral lung transplantation was initiated with continuous VV-ECMO. The procedure itself was extremely challenging owing to severe adhesions resulting from previous thoracotomy, inflammation, infection, and intrapulmonary hemorrhage. The operative time for the transplantation was about 19 h. Currently, at 2 years 8 months postoperatively, the patient is alive and well.

**Conclusion:**

Transplant surgery in this patient was extremely challenging because of the presence of severe pleural adhesions and stiff native lungs secondary to hemorrhagic complications due to the prolonged ECMO support. Surgeons must recognize that lung transplantation after long-term ECMO bridging can be technically more complicated and challenging than shorter-term ECMO.

## Background

Extracorporeal membrane oxygenation (ECMO) has been increasingly used as a bridge to lung transplantation because it stabilizes the recipient with end-stage respiratory failure during the waiting time. Worldwide, most patients undergo transplantation within a few weeks after ECMO initiation [1, 2]. In Japan, however, the waiting period is considerably longer because of a shortage of donors. Furthermore, there are no provisions for prioritizing patients on ECMO [3]. We report a rare, successful case of bilateral lung transplantation in a recipient who had been undergoing veno-venous (VV)-ECMO for 5 months. The most recent follow-up evaluation, at 2 years 8 months postoperatively, revealed that the patient was alive and well with no complaints.

## Case presentation

**Fig. 1:**
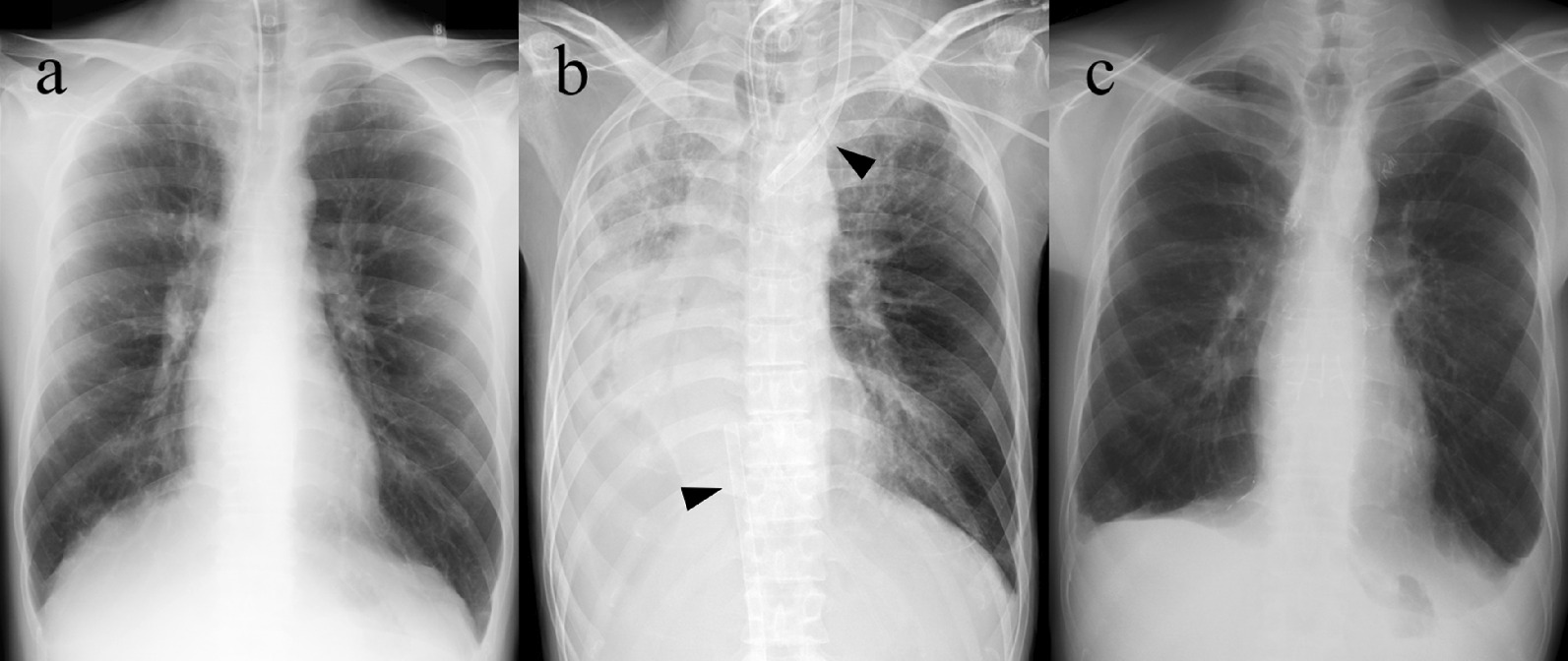
**a** Chest radiographs of the 27-year-old man with bronchiolitis obliterans caused by graft-versus-host disease after bone marrow transplantation. **b** Abnormal chest radiograph with consolidation due to pneumorrhagia in the right lung after long-term veno-venous extracorporeal membrane oxygenation. Two cannulas in the veins (arrowheads) and infiltration of the right lung are seen. **c** Chest radiograph at 18 months after bilateral lung transplantation

A 27-year-old man was referred to our hospital for lung transplantation evaluation. His history included acute myeloid leukemia at age 20 years, for which he underwent bone marrow transplantation (BMTx) 1 year after the diagnosis with fully matched human leukocyte antigen typing. Three years later, he reported dyspnea on exertion and was diagnosed with bronchiolitis obliterans caused by chronic graft-versus-host disease (Fig. 1a). He suffered from invasive pulmonary aspergillosis during his treatment with high-dose inhaled steroids, bronchodilators, and antimicrobial drugs. Home oxygen therapy was initiated because of type II respiratory failure. Four years after BMTx, his condition worsened, requiring mechanical ventilation. He was eventually discharged home with a tracheostomy and mechanical ventilation. At the time of referral back to our hospital, his condition was relatively stable, and he could undertake moderate activities of daily living. He was fully committed to his rehabilitation. A multidisciplinary transplantation selection committee at our hospital deemed him suitable for lung transplantation. After obtaining his consent, we enrolled him on Japan’s national lung-transplantation list.

**Fig. 2:**
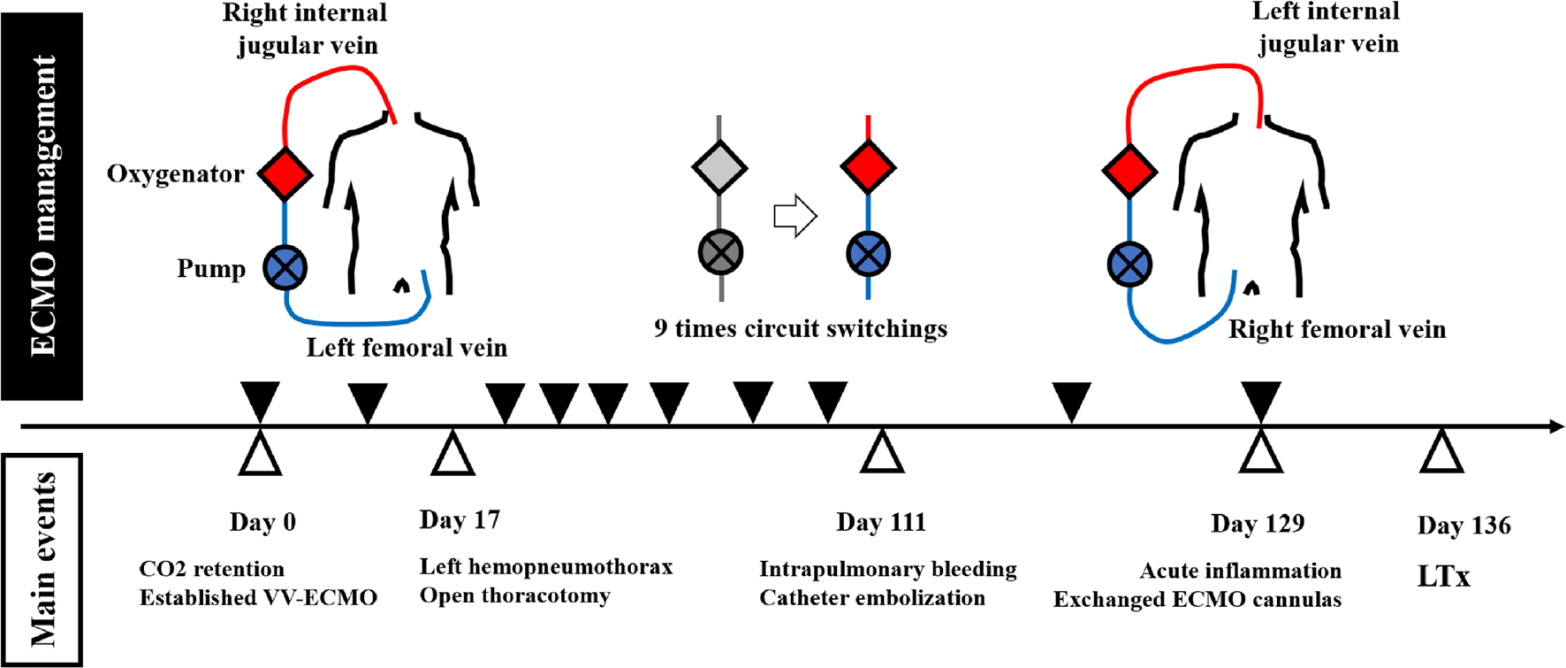
Time course during veno-venous extracorporeal membrane oxygenation before lung transplantation (LTx)

After about a year at home, he developed a right pneumothorax that eventually required thoracotomy. Four months later, he developed a left pneumothorax, which exacerbated carbon dioxide retention. Although the pneumothorax was managed with chest drainage and pleurodesis, the carbon dioxide retention and resulting acidosis were barely manageable with conventional mechanical ventilation, which ultimately necessitated VV-ECMO. ECMO was established via the right internal jugular vein (using a 23-Fr cannula) and the left femoral vein (with a 21-Fr cannula) (Fig. 2), after which he was transferred to an ECMO center at Nippon Medical School Hospital to await lung transplantation.

About 2 weeks later, he developed another left hemopneumothorax. Chest tube drainage resulted in a massive hemothorax, necessitating open thoracotomy for hematoma removal and hemostasis. He attained relative stability despite a few septic episodes caused by *Bacillus cereus*.

**Fig. 3:**
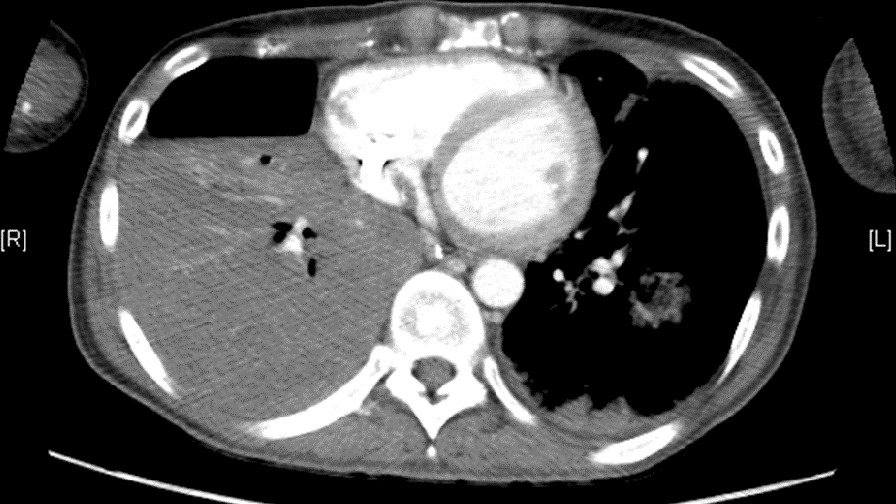
Preoperative computed tomography reveals pneumorrhagia in the right lung

**Fig. 4:**
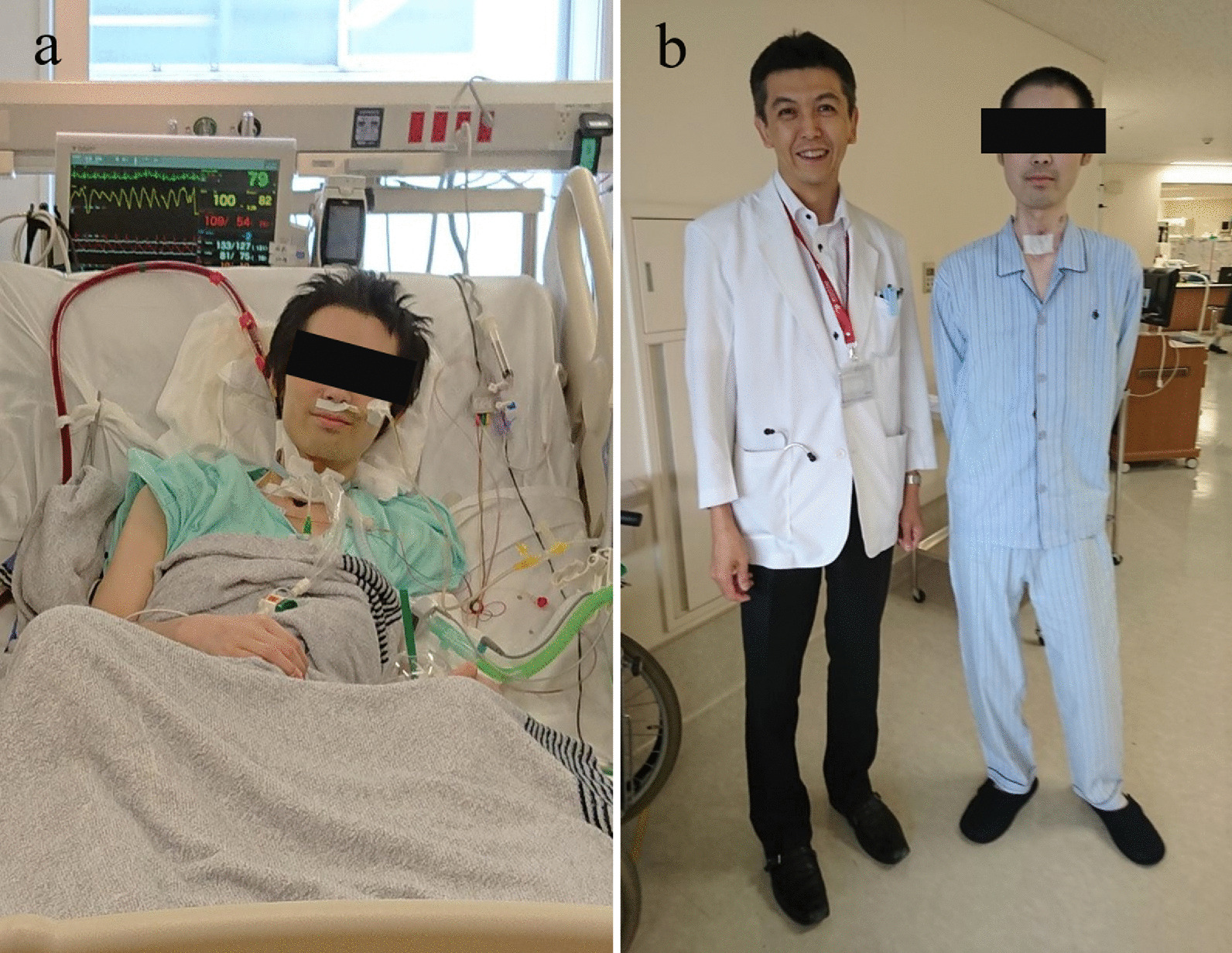
Lung transplant recipient before and after lung transplantation. **a** Four months after initiation of veno-venous extracorporeal membrane oxygenation (before lung transplantation). **b** One year after lung transplantation

On day 111 of ECMO, however, he developed hemoptysis. Computed tomography revealed intrapulmonary bleeding in the right lung (Fig. 3), requiring catheter embolization of the right bronchial artery. On day 129 of ECMO, there were signs of acute inflammation, including an elevated C-reactive protein level (25 mg/dL). Although blood cultures remained negative, the ECMO cannulas were moved to the left jugular vein (19-Fr cannula) and right femoral vein (23-Fr cannula) (Fig. 1b). On day 136 on ECMO, with his general condition continuing to deteriorate, a suitable donor was identified, and the patient was readmitted to our hospital. During the pre-transplant waiting time on VV-ECMO, he had been treated with continuous unfractionated heparin. His activated partial thromboplastin time (APTT) was maintained at around 40–60 s. When bleeding occurred, the APTT was controlled at around 35–40 s. His activated clotting time was also monitored and maintained within the range of 160–200 s. After stabilization, he could eat and drink to some extent but still required mechanical ventilation (Fig. 4a). His rehabilitation was limited because a double-lumen cannula for VV-ECMO was not available in Japan.

At admission, enhanced computed tomography had revealed limited access to the patient’s major veins because of previous cannulation. We therefore consulted with a cardiologist, who inserted a Swan–Ganz catheter through the left subclavian vein (chosen because it appeared to be the only accessible major vein) preoperatively.

Bilateral lung transplantation began with continuous VV-ECMO. Severe adhesions in both thoraces made the procedure challenging. In addition to his history of bilateral thoracotomy, his right lung was inflamed and hard, making it impossible to collapse and difficult to mobilize. After dissecting as much of the intrathoracic adhesions as possible, we switched to veno-arterial ECMO by cannulating the ascending aorta, using two already existing venous cannulas for drainage. Standard pneumonectomy and graft anastomosis were performed with a reasonable ischemia time (left lung: total ischemia 8 h 21 min, warm ischemia 42 min; right lung: total ischemia 11 h 50 min, warm ischemia 52 min). Total blood loss was 34,930 mL.

After reperfusion of the bilateral lungs, we planned to maintain central veno-arterial ECMO because of the massive bleeding and transfusion, the long-term ECMO, and the patient’s unstable preoperative condition. However, the diffuse intrathoracic bleeding was so difficult to control that a half-dose of protamine was administered. Although it resulted in reasonable hemostasis, the membranous oxygenator clotted, with suddenly decreased ECMO flow. Fortunately, the patient’s vital signs, including oxygenation, remained stable despite the diminishing ECMO support.

His postoperative course was uneventful, and 94 days after transplantation he was transferred elsewhere for rehabilitation. He has remained in good condition for 2 years 8 months since the lung transplantation without significant complications (Figs. 1c, 4b). The long-term non-ambulatory VV-ECMO therapy, however, seems to have interfered with his left leg movement, although it is slowly improving.

## Discussion and conclusions

The average waiting time for a lung transplant in Japan is > 800 days (longer than in most Western countries) because of donor shortage [3], and many Japanese patients die from deteriorated respiratory function during this time. Even if they survive the waiting period, their condition has deteriorated to a stage in which the patient is no longer considered suitable for transplantation. We believe that the duration of the VV-ECMO bridging to successful bilateral lung transplantation of our patient is one of the longest so far reported [4]. The patient would never have survived until lung transplantation without the long-term ECMO. It is noteworthy that highly specialized care was required to sustain the patient’s condition and to overcome multiple crises during the extended ECMO support.

Infection and coagulopathy were the two major issues. Hemorrhagic complications during ECMO also posed a serious surgical challenge because of the severe pleural adhesions and immobile, stiff lungs that had developed. Moreover, the long-term ECMO support exhausted the use of accessible central veins, so the anesthetists had to use central veins for access during the transplantation. Although ambulatory ECMO using a double-lumen cannula is ideal, it is debatable whether the cannula is suitable for such long-term ECMO support for bridging to lung transplantation.

In conclusion, we successfully performed bilateral lung transplantation after 5 months of VV-ECMO support. Nevertheless, transplant surgeons must recognize that lung transplantation after long-term ECMO bridging can be technically more complicated and challenging than shorter-term ECMO.

## Data Availability

Not applicable.

## Abbreviations

APTT: Activated partial thromboplastin time
BMTx: Bone marrow transplantation
ECMO: Extracorporeal membrane oxygenation
VV-ECMO: Veno-venous ECMO
